# Relationship between the more-affected upper limb function and daily activity performance in children with cerebral palsy: a cross-sectional study

**DOI:** 10.1186/s12887-021-02927-2

**Published:** 2021-10-19

**Authors:** Hyerin Park, Ja Young Choi, Sook-hee Yi, Eun Sook Park, Dain Shim, Tae Young Choi, Dong-wook Rha

**Affiliations:** 1 Department and Research Institute of Rehabilitation Medicine, Yonsei University College of Medicine, 50-1, Yonsei‑ro, Seodaemun‑gu, Seoul, Republic of Korea; 2 Department of Rehabilitation Medicine, Chungnam National University College of Medicine, 282, Munhwa-ro, Jung-gu, Daejeon, Republic of Korea; 3 Department of Rehabilitation Medicine, Seoul Rehabilitation Hospital, 30, Galhyeon-ro 11-gil, Eunpyeong-gu, Seoul, Republic of Korea

**Keywords:** Cerebral palsy, Activities of daily living, Pediatric rehabilitation

## Abstract

**Background:**

There are differences in roles between the more-affected and less-affected upper limb of children with cerebral palsy (CP). However, there is a lack of studies of the relationship between the more-affected limb function and activities of daily living (ADL) in children with CP. Thus, the aim of this prospective cross-sectional study was to investigate the relationship between more-affected upper limb function and ADL in children with CP.

**Methods:**

Children with spastic CP (unilateral CP *n* = 28, bilateral CP *n* = 31; 34 males, 25 females; mean age ± SD, 6.8 ± 3.1y [range, 3-14y]) participated in this study. Function of the more-affected upper limb was measured using the Melbourne Assessment of Unilateral Upper limb Function, version 2 (MA2) and the Upper Limb Physician’s Rating Scale (ULPRS). Performance of daily living activities was measured using the Pediatric Evaluation of Disability Inventory-Computer Adaptive Test (PEDI-CAT).

**Results:**

The range, accuracy and fluency dimension of MA2 and ULPRS total scores were moderately correlated with the daily activity domain (r = 0.47, 0.47, 0.56 for MA2 and r = 0.50 for ULPRS, respectively; *P* < 0.001) rather than the mobility, social/cognitive, and responsibility domains of the PEDI-CAT. ULPRS scores for elbow extension, supination in extension, supination in flexion, and two-handed function were moderately correlated with the PEDI-CAT daily activity domain (r = 0.44, 0.43, 0.41, and 0.49, respectively; *P* < 0.01). Finger opening and thumb-in-palm deformity of the ULPRS did not correlate with any PEDI-CAT domain.

**Conclusions:**

The MA2 range, accuracy, and fluency domains (rather than dexterity) had the strongest correlations with the PEDI-CAT daily activity domain. Elbow extension, forearm supination, and two-handed function (rather than wrist and finger movements) of the ULPRS had the strongest correlations with the PEDI-CAT daily activity domain.

**Supplementary Information:**

The online version contains supplementary material available at 10.1186/s12887-021-02927-2.

## Background

Children with cerebral palsy (CP) commonly have impairment of upper limb function, with various studies reporting a prevalence ranging from 57 to 83% [1, 2]. Children with spastic CP can be classified into unilateral or bilateral distribution, and both groups frequently display upper limb function impairment [2].

There is often an asymmetry in upper limb function in children with bilateral or unilateral CP, with one limb more severely affected than the other [3, 4]. Most activities of daily living (ADL) are bimanual, but the division of labor may be different for the more-affected and less-affected limbs. In typically developing children, the dominant hand is used for precision tasks, and the non-dominant hand is used for stabilizing tasks [5]. For example, when writing, a child will grip a pencil with the dominant hand and hold the paper with the other. Similarly, as children with CP prefer to use their less-affected limb for ADL, we expect to observe differences in the functional roles between the more-affected and less-affected limbs; the more-affected limb used as the non-dominant hand, and the less-affected limb used as the dominant hand.

A number of interventions (e.g., neuromuscular electrical stimulation, botulinum toxin injections, occupational therapy, orthoses) for the upper limb have been reported in children with unilateral and bilateral CP [6, 7]. The aims of interventions targeting upper limb function in children with CP are to improve functional abilities and increase independence in ADL [6]. The ADL refer to the ability to perform self-care and daily activities, corresponding to activity and participation components of the International Classification of Functioning, Disability, and Health (ICF) [8]. The ICF provides a framework for measuring dysfunction according to level of impairment (structural), activity limitations (difficulty in task execution), and participation restrictions (degree of activity involvement).

Little is known about the relationship between the function of the more-affected limb and ADL in children with CP, although previous studies report a significant relationship between self-care activities and overall manual ability [9–12]. Impaired upper limb function can restrict ADL participation in young adults with CP [13], but this study only analyzed the relationship between ADL and overall upper limb function measured by the Manual Ability Classification System (MACS) and did not evaluate the function of the more-affected upper limb. One report examined the relationship between affected limb function and ADL in unilateral CP, although the approach to ADL classification was simplistic, the participation-restriction category was not examined [12], and no information was provided for how the movement patterns of the affected limbs influenced the performance of ADL.

This study addressed two questions. First, are there any relationships between the four Pediatric Evaluation of Disability Inventory-Computer Adaptive Test (PEDI-CAT) domains (daily activities, mobility, social/cognitive, responsibility) and more-affected limb function in children with spastic CP? Second, if these relationships are identified, which movement patterns of the more-affected limb are associated with ADL performance?

## Methods

### Participants

This cross-sectional study was conducted at three pediatric rehabilitation institutions in the Republic of Korea from July 2017 to March 2018. The study included 59 children with spastic CP with upper limb dysfunction who met the inclusion and exclusion criteria: unilateral CP, *n* = 28; bilateral CP, *n* = 31; 34 males and 25 females; mean age 6 years 8 months, SD 3 years 1 month). The two inclusion criteria were as follows: (1) actively use their arm with manual ability classification system (MACS) levels I to IV and House Functional Classification (HFC) levels 4 to 7, (2) age 3–18 years. The three exclusion criteria were as follows: (1) children with severe mental retardation who could not understand instructions; (2) history of visual impairment; (3) history of operations and/or botulinum toxin injections in an upper limb, or received constraint-induced movement therapy (CIMT) within 6 months of beginning the study. This study was approved by the Institutional Review Boards at all participating hospitals (Severance Hospital, Eulji University Hospital, and Seoul Rehabilitation Hospital). All participants’ parents provided written informed consent. The trial was registered at the Clinical Research Information Service (identifier no. KCT0002395).

### Procedure

One occupational therapist (an author of this study, DS) performed all assessments to exclude inter-rater bias. The Melbourne Assessment of Unilateral Upper Limb Function version 2 (MA2) and the Upper Limb Physician’s Rating Scale (ULPRS) were used to measure limb functions in the more-affected/non-dominant upper limb. The performance of ADL was measured using PEDI-CAT. The MA2 percent score and the PEDI-CAT scaled score were then used for the analyses. In cases of children with bilateral CP, we used data from the non-dominant limb.

### Melbourne assessment of unilateral upper limb function version 2

The MA2 is a well-known tool for measuring unilateral upper limb function in children with neurological impairments aged 2.5–15 years old [14]. The MA2 has 30 possible scores in four dimensions based on 14 tasks of reaching, grasping, releasing, and manipulating objects: (1) range of motion (ROM), (2) accuracy, (3) dexterity, and (4) fluency of upper limb function. MA2 has high inter-rater, intra-rater, and test-retest reliability [intraclass correlation coefficient (ICC) = 0.90–1.00] for the four subscale scores [15], and video is taken during testing for accurate scoring [16]. The scoring system uses a 3-, 4-, or 5-point scale, with lower scores representing worse impairment. A raw score is the sum of each individual score, and the percent score represents the raw score divided by the maximum total score, multiplied by 100 [17]. We computed the percent score for these analyses.

### Upper limb Physician’s rating scale assessment

The ULPRS is a semiquantitative assessment that evaluates movement patterns and determines functional impairment of the upper limb by focusing on all three arm levels, including the palm, forearm, and elbow (Supplemental Table S1) [18]. The advantages of the ULPRS include its ease of use by healthcare providers without the need for special training, and high inter-rater and intra-rater reliability (ICC = 0.94 between raters, and ICC = 0.99–1.00 within raters) [19]. Among other parameters, the ULPRS assessment can determine whether there is an isolated functional impairment, such as restricted forearm supination, a wrist in flexion and deviation, elbow flexion, or thumb-in-palm, with unilateral limb scores ranging from 0 to 25 (lower scores represent worse impairment).

### Pediatric evaluation of disability inventory-computer adaptive test

The PEDI-CAT is a standardized outcome measure that assesses functional activities in children and young people [20, 21]. The PEDI-CAT is a revised version of the original Pediatric Evaluation of Disability Inventory (PEDI). The original PEDI was used as a comprehensive functional assessment of key activity performance in children aged 6 months to 7.5 years, whereas the PEDI-CAT is recommended for assessing children aged 0 to 21 years with different health conditions. The PEDI-CAT incorporates a computer-adaptive platform with 276 items based on parental or caregiver reporting, and has four domains that cover daily activities, mobility, social/cognitive function, and responsibility [22].

The PEDI-CAT was designed to be consistent with the ICF and corresponds to the activities and participation components of the ICF [23]. The daily activity, mobility, and social/cognitive domains of the PEDI-CAT all correspond to activity dimensions in the ICF (defined by the performance of a variety of tasks), and the PEDI-CAT responsibility domain corresponds to the ICF participation dimension (defined as being involved in real-life situations) [22].

The PEDI-CAT outcome measure has strong reliability and construct validity in children with CP [24]. There are two versions of the assessment: a “speedy” PEDI-CAT, and a content-balanced PEDI-CAT. We used the speedy version because of its efficiency. A scaled score for each domain (ranging from 20 to 80) was used for the analyses (lower scaled scores represent lower functional activities).

### Statistical analysis

Data were analyzed using the Statistical Package for the Social Sciences Program (SPSS version 23.0). The Shapiro-Wilk test was used to test for a normal distribution of the data. Pearson and Spearman correlation coefficients were calculated to estimate correlations between the MA2 scores and each of the four PEDI-CAT domain scaled scores. Spearman correlation coefficients were used to calculate correlations between ULPRS scores and each of the four PEDI-CAT domain scaled scores. We calculated partial correlation coefficients to control for the potential confounders of age and Gross Motor Function Classification System (GMFCS) levels. Correlation coefficient values of < 0.2, 0.2–0.4, 0.4–0.6, 0.6–0.8, and > 0.8 were considered as very weak, weak, moderate, strong, and very strong, respectively [25]. We considered correlation coefficient values > 0.4 as meaningful.

## Results

### Participant characteristics

A total of 59 children with spastic CP (34 males and 25 females) aged 3 to 14 years (mean age 6 years 8 months, SD 3 years 1 month) were included in this study. Twenty-eight children had unilateral CP, and 31 children had bilateral CP. The descriptive characteristics of the participants including age, sex, more-affected side, involved side, and distribution of GMFCS, MACS, and HFC levels are presented in Table 1.

**Table 1:** Participant characteristics

Characteristic	Number/Value^a^
Number of participants	59
Age at assessment	6.8 ± 3.1 (3–14)
More-affected side, right/left	31/ 28
Sex, male/female	34/ 25
Involved side, unilateral/bilateral	28/ 31
GMFCS level, I/II/III/IV	25/19/8/7
MACS level, I/II/III/IV	2/25/18/14
HFC 4/5/6/7	14/15/22/8

GMFCS, Gross Motor Function Classification System; MACS, Manual Ability Classification System; HFC, House Functional Classification

^a^ Values are mean ± SD (range)

### MA2 and PEDI-CAT correlations

**Fig. 1:**
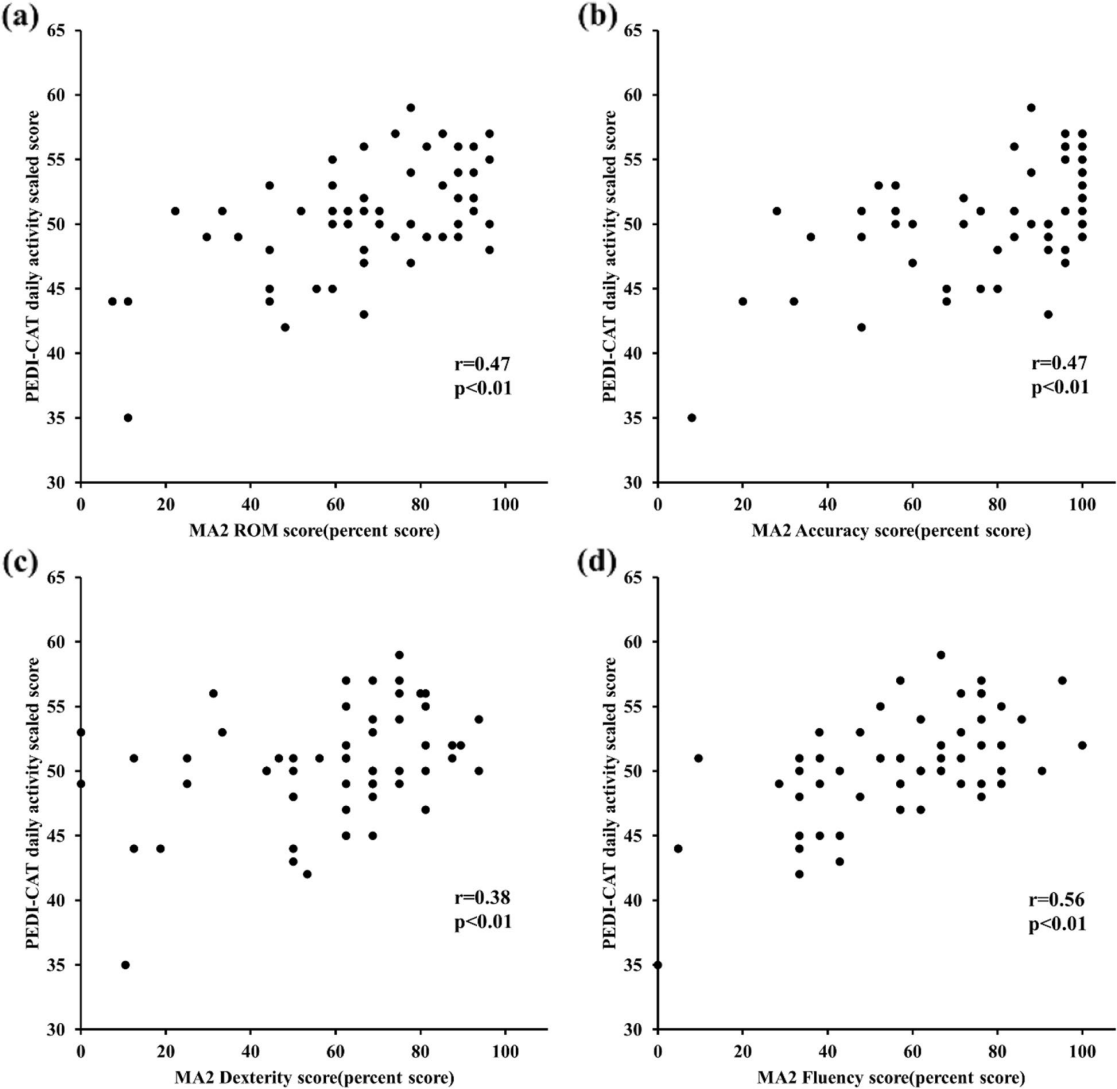
Correlation between MA2 dimensions and PEDI-CAT daily activity score. **a** MA2 ROM percent score correlation with PEDI-CAT daily activity scaled score (r = 0.47, *P* < 0.01). **b** MA2 accuracy percent score correlation with PEDI-CAT daily activity scaled score (r = 0.47, *P* < 0.01). **c** MA2 dexterity percent score correlation with PEDI-CAT daily activity scaled score (r = 0.38, *P* < 0.01). **d** MA2 fluency percent score correlation with PEDI-CAT daily activity scaled score (r = 0.56, *P* < 0.01)

Partial correlations between MA2 scores and PEDI-CAT domains are presented in Table 2. Scatter diagrams of the PEDI-CAT daily activity domain plotted against MA2 range, accuracy, dexterity, and fluency dimension scores are presented in Fig. 1.

**Table 2:** Partial correlation coefficients between the MA2 and the PEDI-CAT

	PEDI-CAT			
	Daily activity	Mobility	Social/cognitive	Responsibility
**MA2**				
Range				
Correlation	0.47^a^	0.26	0.19	0.32
*P*-value	< 0.001^**^	0.056	0.167	0.014^*^
Accuracy				
Correlation	0.47^a^	0.22	0.24	0.30
*P*-value	< 0.001^**^	0.095	0.072	0.022^*^
Dexterity				
Correlation	0.38	0.13	0.12	0.12
*P*-value	0.004^**^	0.355	0.391	0.390
Fluency				
Correlation	0.56^a^	0.40^a^	0.23	0.40^a^
*P*-value	< 0.001^**^	0.002^**^	0.083	0.002^**^

MA2, Melbourne Assessment of Unilateral Upper Limb Function, version 2; PEDI-CAT, Pediatric Evaluation of Disability Inventory-Computer Adaptive Test

Partial correlation coefficients are adjusted by age and GMFCS levels and are Spearman’s r values for daily activity, mobility and Pearson’s r values for social/cognitive, and responsibility domains of the PEDI-CAT

^*^Two-tailed significance, *P* < 0.05

^**^ Two-tailed significance, *P* < 0.01

Significant *P*-values are highlighted in bold font

^a^ Correlation coefficient value 0.4–0.6; moderate correlation

MA2 range, accuracy, and fluency dimensions were moderately positively correlated with the PEDI-CAT daily activity domain (r = 0.47, 0.47, 0.56, respectively; *P* < 0.001), and were weakly to moderately correlated with the PEDI-CAT responsibility domain (r = 0.32, 0.30, 0.40, respectively; *P* < 0.05). The MA2 dexterity dimension was weakly correlated with the PEDI-CAT daily activity domain, whereas it was not significantly correlated with the PEDI-CAT mobility, social/cognitive, and responsibility domains. No MA2 dimension correlated with the PEDI-CAT social/cognitive domain.

### ULPRS and PEDI-CAT correlations

Partial correlations between ULPRS scores and PEDI-CAT domains are presented in Table 3. Active elbow extension, active supination in elbow extension, and active supination in flexion were moderately positively correlated with the PEDI-CAT daily activity domain (r = 0.44, 0.43, and 0.41, respectively; *P* < 0.01). The two-handed function score had the highest correlation coefficient for the PEDI-CAT daily activity domain (r = 0.49; *P* < 0.001). Finger opening and thumb-in-palm were not significantly correlated with the PEDI-CAT daily activity domain. The PEDI-CAT responsibility domain was only weakly correlated with each ULPRS domain, except for finger and thumb movement.

**Table 3:** Partial correlation coefficients between the ULPRS and the PEDI-CAT

	PEDI-CAT			
	Daily activity	Mobility	Social/cognitive	Responsibility
**ULPRS**				
Active elbow extension				
Correlation	0.44^a^	0.32	0.24	0.36
*P*-value	0.001^**^	0.015^*^	0.071	0.006^**^
Active supination in extension				
Correlation	0.43^a^	0.33	0.24	0.30
*P*-value	0.001^**^	0.012^*^	0.070	0.024^*^
Active supination in flexion				
Correlation	0.41^a^	0.40^a^	0.23	0.35
*P*-value	0.002^**^	0.002^**^	0.091	0.009^**^
Active wrist dorsiflexion				
Correlation	0.27	0.13	0.23	0.32
*P*-value	0.045^*^	0.336	0.092	0.015^*^
Wrist deviation				
Correlation	0.36	0.23	0.31	0.38
*P*-value	0.006^**^	0.088	0.019^*^	0.004^**^
Finger opening				
Correlation	0.06	−0.03	0.08	0.15
*P*-value	0.677	0.807	0.562	0.260
Thumb in palm				
Correlation	0.22	−0.04	0.06	0.13
*P*-value	0.107	0.796	0.669	0.344
Associated increase in muscle tone				
Correlation	0.38	0.29	0.05	0.21
*P*-value	0.004^**^	0.026^*^	0.713	0.117
Two-handed function				
Correlation	0.49^a^	0.38	0.25	0.35
*P*-value	< 0.001^**^	0.003^**^	0.059	0.008^**^
Total score				
Correlation	0.50^a^	0.31	0.28	0.42^a^
*P*-value	< 0.001^**^	0.021^*^	0.037^*^	0.001^**^

ULPRS, Upper Limb Physician’s Rating Scale; PEDI-CAT, Pediatric Evaluation of Disability Inventory-Computer Adaptive Test

Partial correlation coefficients are adjusted by age and GMFCS levels and are Spearman’s r values. Significant *P*-values are highlighted in bold font

^*^ Two-tailed significance, *P* < 0.05, ^**^ Two-tailed significance, *P* < 0.01

^a^Correlation coefficient value 0.4–0.6; moderate correlation

## Discussion

The first aim of this study was to examine the relationships between the four PEDI-CAT domains and the functionality of the more-affected limb in children with spastic CP. We identified moderately positive correlations between the PEDI-CAT daily activity domain and the function of the more-affected upper limb in children with CP. There was no significant correlation between the PEDI-CAT social/cognitive domain and the function of the more-affected upper limb in children with CP.

The results indicated that upper limb function in children with CP (assessed using MA2 and ULPRS) was primarily correlated with the PEDI-CAT daily activity domain rather than other domains. Real-life hand skill performance was reported to be a contributing factor of self-care function in children with and without disabilities [26]. Previous research reported a strong correlation between upper-extremity function (assessed using the original Melbourne Assessment) and the PEDI self-care domain [17], which is consistent with our results correlating the PEDI-CAT daily activity domain (equivalent to the original PEDI functional skills of self-care domain) with upper-extremity function. However, the previous study had smaller sample size, did not analyze other ADL domains, and did not specifically target the function of the more-affected limb [17]. Our results showed the importance of improving the function of the more-affected upper limb to enhance daily activities, such as dressing, eating, and grooming. The PEDI-CAT responsibility domain was only weakly correlated with MA2 values for children with CP. MA2 is a tool that measures unimanual capacities, and the PEDI-CAT responsibility domain corresponds with the ICF participation dimension. Therefore, a weak association between MA2 and PEDI-CAT responsibility domain might indicate a gap between participant unimanual capacities and their participation.

We found no significant relationship between upper limb function in children with CP and the PEDI-CAT social/cognitive domain. This domain represents the ability to interact with people, and includes the skills needed for effective social exchanges and to function safely [22]. Social participation impairments in children with CP are related to intellectual function, environmental factors, and motor impairment [27, 28]. Therefore, the PEDI-CAT social/cognitive domain might be affected by a variety of factors. Further studies are needed to examine the factors that affect social and cognitive aspects of behavior in children with CP.

The second aim of this study was to identify the movement patterns of the more-affected limb that were related to the four PEDI-CAT domains in children with CP. We found that there was weak association between dexterity and PEDI-CAT daily activities, compared to moderate correlations between MA2 domains (range, accuracy, and fluency) and PEDI-CAT daily activities. The dexterity test consists of grasp/release of crayon, grasp/release of pellet, and manipulation, all of which are related to finger movement. ULPRS assessments for finger opening and thumb-in-palm were not significantly correlated with the PEDI-CAT daily activity domain. Finger opening and thumb-in-palm are related to articular movements of the hand and fingers, which are considered as fine motor skills. Our results indicated that fine motor skills (smaller movements that occur in the hands and fingers) in the more-affected upper limb were not significantly or weakly correlated with the PEDI-CAT daily activity domain.

Most ADL consist of bimanual activities, and the roles of dominant and non-dominant hands are very different for these everyday activities. Children with CP use the more-affected hand to assist in bimanual activities, and use the less-affected hand for unimanual activities. Differences in function between the more and less-affected hands can lead to their different roles in daily activities.

Children with unilateral CP prefer to use the less-affected hand for precision tasks and the more-affected hand (the assisting hand) for complementary tasks, such as holding and stabilizing [29]. A previous study reported that the more-affected hand was used to provide fixation (usually requires fewer fine motor skills), whereas the less-affected hand was used for manipulation (associated with fine motor skills) [30]. However, these earlier reports only examined assisting hand preferences, and did not provide information about the assisting hand movement patterns that affected ADL. Our present results suggest that the assisting hand movement patterns affecting ADL were more related to complementary tasks than precision tasks.

The functional ULPRS assessment of the more-affected upper limb showed that PEDI-CAT daily activities were more closely related to proximal-joint motions (elbow extension and forearm supination) than to distal-joint motions of the wrist and hand. The fine motor skills in the wrists and hands are related to precision tasks, whereas proximal-joint movements are more relevant for complementary tasks than detailed tasks. Our results showed that ADL was more closely correlated with proximal-joint movements than distal joint movements of the more-affected limb. We propose that more consideration should be given to rehabilitation programs that provide therapy for the more-affected limb to act as an assisting hand for performing complementary tasks rather than performing precision tasks, which could improve ADL in children with CP.

Klingels et al. [31] described the association between distal upper limb impairments (distal muscle strength and grip strength) and activity measures. Mailleux et al. [32] reported the importance of distal limb movements for the execution of functional tasks. These discrepancies might be explained by the wide age ranges and severity of upper limb function impairment in the studies. Further studies are needed to verify these results, such as a comparative analysis of the effects of botulinum toxin injections on elbow flexors and pronators versus injections into wrist flexors and thumb adductors.

Another important finding was that ULPRS two-handed function scores were moderately correlated with the PEDI-CAT daily activity domain. Bimanual performance is significantly associated with ADL motor skills in unilateral CP [12], and a strong positive association between self-care and bimanual performance was reported [33]. Previous CIMT and Hand Arm Bimanual Intensive Training (HABIT) studies also revealed the importance of bimanual training for improving ADL in children with CP [34, 35]. CIMT and HABIT are frequently used as interventions for upper limb training in children with CP. CIMT restrains the non-affected limb to promote the use of the affected limb, and HABIT is an intensive treatment that provides bimanual activities to improve bimanual coordination. CIMT has achieved greater efficacy than HABIT in improving impaired arm function in children with hemiplegic CP, although HABIT achieved greater improvement in bimanual performance and ADL than CIMT [34, 35]. Considering our results and those of previous studies, it might be helpful to implement bimanual and unimanual training to improve ADL, and two-handed function should be assessed before and after any intervention for the more-affected limb.

A limitation of this study was that the more-affected limb function was primarily assessed during unimanual activities, so further studies are needed to use existing tools to evaluate the function of the affected limb during bimanual activities. For example, the Assisting Hand Assessment (AHA) can be performed to measure the effectiveness of the assisting hand in bimanual activities of children with unilateral impairment [29]. The Both Hands Assessment (BoHA) can be used to measure the effectiveness of bimanual performance and the extent of asymmetric hand use in children with bilateral cerebral palsy [36]. The correlations between upper limb function and the PEDI-CAT daily activity domain were not strong; therefore, follow-up studies on other factors affecting ADL are needed. As this study was a cross-sectional design, the direction of causality between the function of the more-affected upper limb and daily activity performance remains unclear, and the results may not be generalizable. Nevertheless, we believe our results provide useful insights into upper limb interventions to improve ADL. Further studies to investigate whether rehabilitation management for upper limb training for the assisting hand during bimanual activities can enhance ADL, which may help to clarify any relationships between the affected upper limb function and the performance of ADL in children with CP.

## Conclusions

This study demonstrated that the functionality of the more-affected upper limb (assessed by MA2 and ULPRS) correlated with the performance of ADL in children with spastic CP. MA2 and ULPRS assessments were significantly correlated with the PEDI-CAT daily activity domain, but not with the PEDI-CAT social/cognitive domain. The MA2 range, accuracy, and fluency dimensions (rather than dexterity), and the elbow extension, forearm supination, and two-handed function (rather than wrist and finger movements) assessed by the ULPRS displayed the strongest correlations with the PEDI-CAT daily activity domain.

## Supplementary Information

**Additional file 1: Supplemental Table S1.** Upper Limb Physician’s Rating Scale. Upper Limb Physician’s Rating Scale.

## Data Availability

The data sets generated and analyzed during the current study are not publicly available due to the privacy of research participants, but are available from the corresponding author on reasonable request.

## Abbreviations

CP: cerebral palsy
MA2: Melbourne Assessment of Unilateral Upper Limb Function version 2
GMFCS: Gross Motor Function Classification System; MACS, Manual Ability Classification System
PEDI-CAT: Pediatric Evaluation of Disability Inventory-Computer Adaptive Test
ULPRS: Upper Limb Physician’s Rating Scale
CIMT: Constraint-Induced Movement Therapy
HABIT: Hand Arm Bimanual Intensive Training
AHA: Assisting Hand Assessment
BoHA: Both Hands Assessment
