# Supplementary material for: Relationship between the more-affected upper limb function and daily activity performance in children with cerebral palsy: a cross-sectional study
Source: BMC Pediatr. 2021 Oct 19;21:459. doi: 10.1186/s12887-021-02927-2 (PMC8524797; doi:10.1186/s12887-021-02927-2)
Supplement: Supplementary file 1 — Additional file 1: Supplemental Table S1. Upper Limb Physician’s Rating Scale. Upper Limb Physician’s Rating Scale. [file 12887_2021_2927_MOESM1_ESM.docx]

Relationship between the more-affected upper limb function and daily activity performance in children with cerebral palsy: a cross-sectional study

Hyerin Park^1^, Ja Young Choi^2^, Sook-hee Yi^3^, Eun Sook Park^1^, Dain Shim^1^, Tae Young Choi^1^ and Dong-wook Rha^1^

^1^Department and Research Institute of Rehabilitation Medicine, Yonsei University College of Medicine, 50-1, Yonsei‑ro, Seodaemun‑gu, Seoul, Republic of Korea

^2^ Department of Rehabilitation Medicine, Chungnam National University College of Medicine, 282, Munhwa-ro, Jung-gu, Daejeon, Republic of Korea

^3^ Department of Rehabilitation Medicine, Seoul Rehabilitation Hospital, 30, Galhyeon-ro 11-gil, Eunpyeong-gu, Seoul, Republic of Korea

* Correspondence:

Dong-wook Rha, MD, PhD.

Department and Research Institute of Rehabilitation Medicine

Yonsei University College of Medicine

50-1, Yonsei-ro, Seodaemun-gu, Seoul, Republic of Korea

Tel +82-2-2228-3717

E-mail address: medicus@yuhs.ac.

**Supplemental Table S1.** Upper Limb Physician’s Rating Scale

| Parameter | Definition | Score |
| --- | --- | --- |
| Active elbow extension (normal 180°) | >10° reduction | 0 |
|  | 1–10° reduction | 1 |
|  | No reduction | 2 |
| Active supination in extension (elbow extended, forearm supinates) Mid-position: palm 90° to horizontal | None | 0 |
|  | Under mid-position | 1 |
|  | To mid-position | 2 |
|  | Past mid-position | 3 |
| Active supination in flexion  (elbow flexed 90°, forearm supinates) | None | 0 |
|  | Under mid-position | 1 |
|  | To mid-position | 2 |
|  | Past mid-position | 3 |
| Active wrist dorsiflexion  (forearm supported, active dorsiflexion of wrist).  Mid-position: palm level with forearm | None | 0 |
|  | Under mid-position | 1 |
|  | To mid-position | 2 |
|  | Past mid-position | 3 |
| Wrist dorsiflexion   (angle of movement) | With ulnar deviation | 0 |
|  | With radial deviation | 0 |
|  | Neutral | 1 |
| Finger opening | Only with wrist flexion | 0 |
|  | With wrist in neutral position | 1 |
|  | With wrist in dorsiflexion | 2 |
| Thumb in function | Within palm | 0 |
|  | Pressed laterally against index finger | 1 |
|  | Partly assists in grasp | 2 |
|  | Thumb-finger grasp possible | 3 |
|  | Active abduction | 4 |
| Associated increase in muscle tone | In all manipulative function | 0 |
|  | Only with fine motor manipulation | 1 |
|  | Only with walking or running | 2 |
|  | None | 3 |
| Two-handed function | None | 0 |
|  | Poor, no use of hidden functions | 1 |
|  | Use of all functions, but limited in ADL | 2 |
|  | Use of all functions, not limited in ADL | 3 |
| Total score |  | 47 |

Adapted from Graham HK, et al. Gait Posture 2000;11:67-79.
